# Hysteroscopic management of a heterotopic pregnancy following uterine artery embolization: a case report

**DOI:** 10.1186/s13256-016-1109-y

**Published:** 2016-11-15

**Authors:** Jigyasa Subedi, Min Xue, Xin Sun, Dabao Xu, Xinliang Deng, Kenan Yu, Xi Yang

**Affiliations:** Department of Obstetrics and Gynecology, Third Xiangya Hospital of Central South University, Changsha, Hunan 410013 China

**Keywords:** Cervical pregnancy, Intra-uterine pregnancy, Heterotopic pregnancy, Hysteroscopy, Surgical management, Uterine artery embolization, Case report

## Abstract

**Background:**

Intra-uterine pregnancy coexisting with cervical pregnancy (heterotopic pregnancy) is a rare condition and its management is challenging because of the massive bleeding associated with cervical pregnancy. Uterine artery embolization followed by hysteroscopic removal of cervical and intra-uterine products of conception can theoretically prevent massive bleeding and provide a direct view during the removal. Hysteroscopic management following uterine artery embolization of heterotopic pregnancy after in vitro fertilization and embryo transfer is rarely reported.

**Case presentation:**

A 33-year-old primigravida, Asian, married, nonsmoker, nonalcoholic woman presented with heavy vaginal bleeding 3 weeks after in vitro fertilization and embryo transfer with a documented history of two embryo implantations in her uterine cavity. Transvaginal ultrasonography revealed a gestational sac of 15 mm × 9 mm × 9 mm with cardiac activity, showing a 3.0-mm-diameter yolk sac in the uterine cavity and a 15 mm × 11 mm × 8 mm gestational sac with cardiac activity, showing a 2.9-mm-diameter yolk sac in the cervical canal. The bilateral uterine artery embolization followed by hysteroscopic removal of both the gestational products was successfully performed after our patient and her family chose to give up the intra-uterine pregnancy due to the risk of heavy bleeding associated with cervical pregnancy.

**Conclusions:**

Uterine artery embolization followed by hysteroscopic removal of cervical and intra-uterine gestational products in the first trimester is safe and feasible, while preserving future fertility.

## Background

Ectopic pregnancy is an abnormal gestation in which the fertilized ovum is implanted outside the uterine cavity and the ampulla region of fallopian tube is the most common site of implantation [1]. In vitro fertilization and embryo transfer (IVF-ET) has been described as one of the major causes for the condition [2]. The incidence of multiple pregnancy has also increased with the increased use of assisted reproductive technology (ART) and it complicates approximately 0.8 % of pregnancies following infertility treatment [3]. Cervical pregnancy is a life-threatening condition accounting for less than 1 % of ectopic gestations ranging between 1:10^3^ and 1:18 × 10^4^ pregnancies [4]. It generally presents with profuse and painless vaginal bleeding, so diagnosis based on ultrasound imaging should be done as early as possible in order to facilitate the management for controlling severe vaginal bleeding [5, 6]. There are several case reports in the literature about the successful usage of methotrexate administration either intra-muscularly or intra-amniotically, ultrasound-guided injection of potassium chloride (KCl) into the cervical gestation sac or the combination of both methotrexate and KCl injections [7], and uterine artery embolization (UAE) [8]. In any of the cases, close follow-up is mandatory in order to ensure the efficacy of the applied therapeutics and may demand additional interventions such as dilatation and curettage, hysteroscopic resection, and even hysterectomy. Thus there is no consensus regarding the best therapeutic approach for cervical pregnancy. The incidence of heterotopic pregnancy is extremely low, and a review of available literature fails to find a specific protocol for the management of such a condition. It is well established that hysteroscopy can provide a direct view [9], and uterine artery embolization can prevent heavy uterine bleeding. With this in mind, we have successfully carried out hysteroscopic removal of gestational products of a heterotopic pregnancy following uterine artery embolization. To the best of our knowledge, this is the first case report about such a management for heterotopic pregnancy after IVF-ET.

## Case presentation

A 33-year-old primigravida, Asian, married, nonsmoker, nonalcoholic woman presented with a 3-week history of IVF-ET with documented history of two embryo implantations and 3-day history of painless vaginal bleeding to our outpatient department in May, 2015. The bleeding had increased in volume and she had passed an increasing number of clots over 1 day prior to the presentation. She had undergone right adnexectomy for a right ovarian cyst 1 year ago. She had no history of infectious diseases including sexually transmitted diseases. She had no significant family history including any inherited disorders. She belonged to the middle socioeconomic class.

**Fig. 1:**
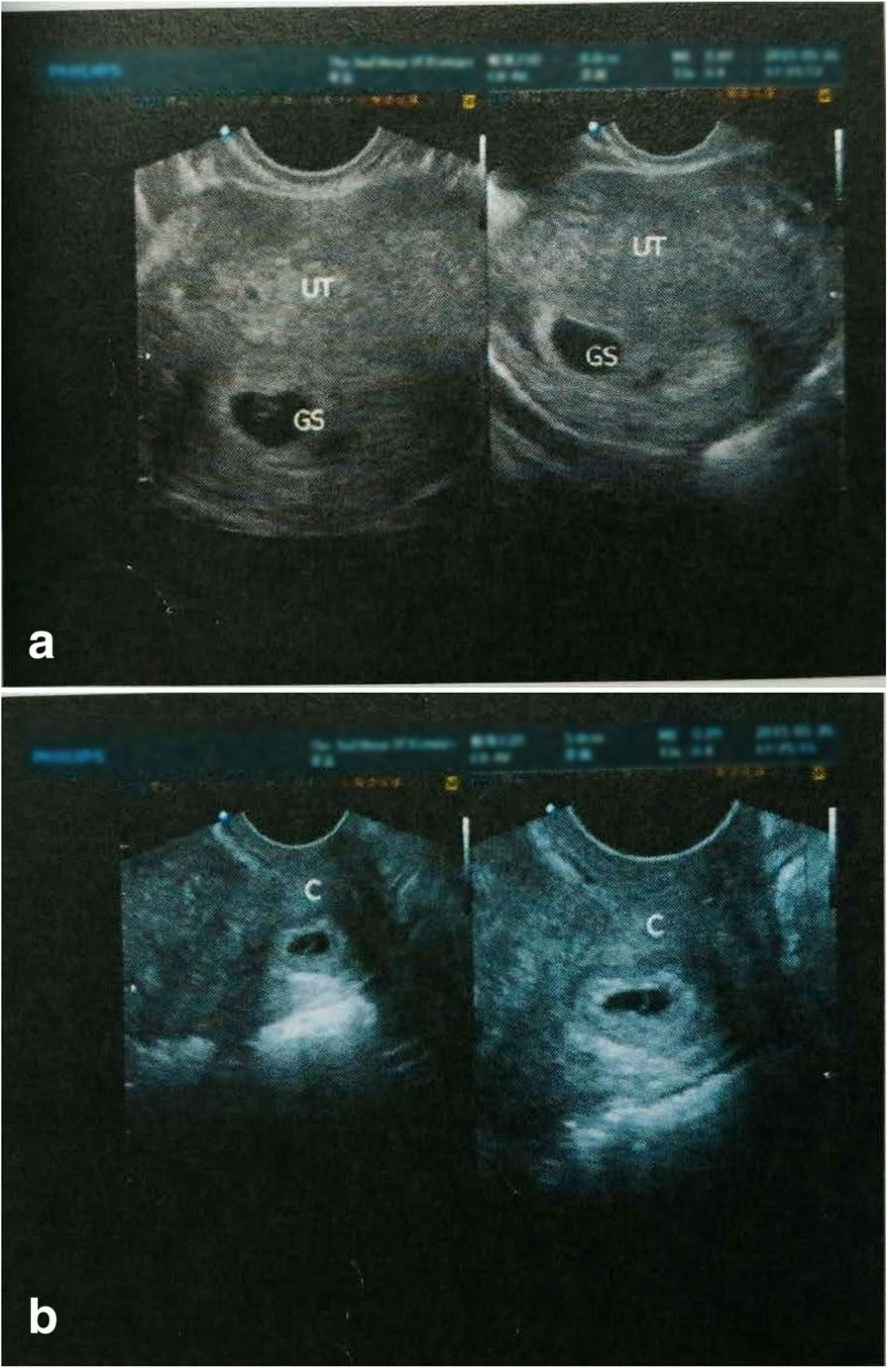
**a** Transvaginal Ultrasonography showing a gestational sac in the uterine cavity. GS = Gestational Sac, UT = Uterus. **b** Transvaginal Ultrasonography showing a gestational sac in the cervical canal. C = cervical canal.

On examination, she was conscious, cooperative, and well-oriented with stable vitals. Per abdominal examination revealed mild tenderness in the lower abdomen. Her quantitative beta-human chorionic gonadotropin (β-hCG) level was 9000 mIU/L. Transvaginal ultrasonography revealed an anterior uterus (56 mm × 45 mm × 52 mm). A gestational sac (15 mm × 9 mm × 9 mm) with cardiac activity, and a 3.0-mm-diameter yolk sac was located in her uterine cavity (Fig. 1a). The cervical length was 43 mm. A second gestational sac (15 mm × 11 mm × 8 mm) with cardiac activity, showing a 2.9-mm-diameter yolk sac was located in her cervix (Fig. 1b). There was a 7 mm × 6 mm × 6 mm hypoechoic nodule (blood clot) at the bottom of her uterus. Her left ovary was normal.

**Fig. 2:**
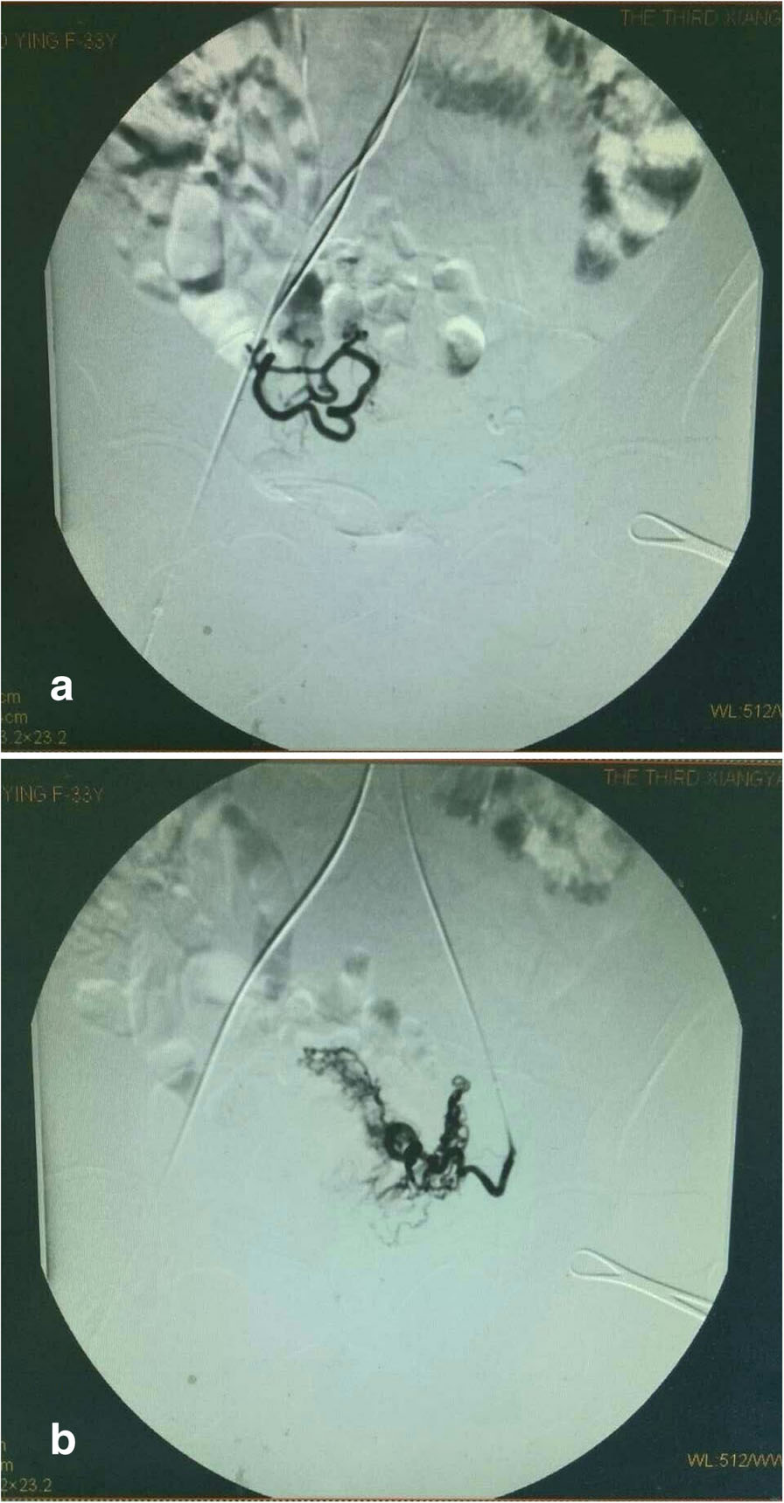
**a** and **b** Hysteroscopic images after bilateral uterine artery embolization

**Fig. 3:**
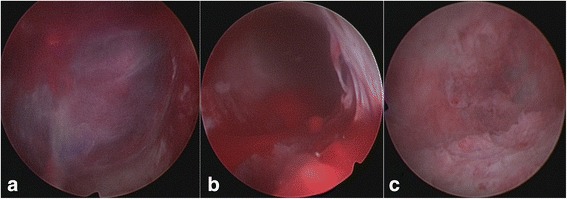
**a** Hysteroscopic image of an undifferentiated embryo in the cervical canal suggesting cervical pregnancy. **b** Hysteroscopic image immediately after the curettage of the products of conception in the uterus. **c** Hysteroscopic image after completion of the curettage procedure showing the uterine cavity

Our patient was admitted with the diagnosis of heterotopic pregnancy. Her hemoglobin level was 110 g/L and leukocyte count was 1 × 10^9^/L. She had continuous vaginal bleeding even after admission. Our patient and her family did not give consent for continuing the intra-uterine pregnancy due to risk of massive bleeding associated with cervical pregnancy. A bilateral UAE was done with an intention of controlling uterine bleeding and preserving the uterus (Fig. 2a and b). Two days after the UAE, hysteroscopy was proposed for our patient. Intra-operative findings included an enlarged cervical canal and the discovery of 2 cm × 3 cm × 2 cm and 2 cm × 2 cm × 2 cm dark red tissue in the posterior wall of her cervical canal and anterior wall of her uterine cavity, respectively. Removal of both the gestational products using vacuum suction followed directly by hysteroscopic removal using 7 Fr forceps was performed successfully. After the surgery, there were no retained gestational products inside her cervical canal or intra-uterine cavity. Intra-operative and postoperative bleeding were minimal (Fig. 3a–c). Histopathology reports confirmed the presence of chorionic villi and decidual tissue in both gestational products.

Our patient was doing well after surgery and her β-hCG titer level was normal 4 weeks postoperatively. Our patient’s vaginal bleeding stopped 1 week after the surgery.

## Discussion

With the increased use of ART, there is an increased rate of multiple pregnancies. Among patients with tubal factor infertility, the incidence of IVF ectopic pregnancy varies from 1.8 to 11 % [10]. Among the ectopic pregnancies achieved through assisted reproductive technologies, cervical pregnancy accounts for 3.7 % of IVF-ET [11].

The management of cervical pregnancy is challenging due to the associated life-threatening complications [12, 13]. The most effective mode of treatment is still controversial, but the patient can be managed either conservatively or radically. Conservative management includes medical or surgical treatments. Medical treatments include intra-amniotic or systemic administration of methotrexate alone or in combination with KCl for intra-amniotic injection [7]. There is, however, the possibility that when using these approaches the products of gestation may not be resolved or evacuated completely and may remain inside the canal for an extended duration, thus necessitating additional interventions.

Surgical procedures include ultrasound-guided aspiration, cervical tamponade, internal iliac artery or uterine artery ligation or embolization, hysteroscopy-guided curettage, and cervical cerclage [14]. The main problem with various conservative treatments is life-threatening hemorrhage which may necessitate hysterectomy as the radical approach. But in our case, the patient and her family did not give consent for continuing the intra-uterine pregnancy. Our patient and her family were afraid of the possible heavy bleeding associated with cervical pregnancy and any side effects to the fetus by the treatment of the cervical pregnancy. That is why they chose to give up the intra-uterine pregnancy. Therefore, uterine artery embolization was performed in order to control vaginal bleeding due to cervical pregnancy, and to preserve her uterus for future fertility. The subsequent hysteroscopic resection of the retained products of conception was performed, confirming that no retained products of conception were left behind inside her uterus and cervical canal [9]. Our patient recovered well after the surgery.

## Conclusions

In conclusion, for the population with heterotopic pregnancy in the first trimester and whose future fertility needs to be preserved, hysteroscopic removal of both gestational products following UAE is an ideal alternative management because it can prevent massive uterine bleeding, and hysteroscopy can provide a direct view for removal of the retained gestational products after vacuum suction.
